# Patient-specific brain arteries molded as a flexible phantom model using 3D printed water-soluble resin

**DOI:** 10.1038/s41598-022-14279-7

**Published:** 2022-06-17

**Authors:** Daniel P. G. Nilsson, Madelene Holmgren, Petter Holmlund, Anders Wåhlin, Anders Eklund, Tobias Dahlberg, Krister Wiklund, Magnus Andersson

**Affiliations:** 1 Department of Physics, Umeå University, 901 87 Umeå, Sweden; 2 Department of Radiation Sciences, Radiation Physics, Biomedical Engineering, Umeå University, 901 87 Umeå, Sweden; 3 Department of Clinical Science, Neurosciences, Umeå University, 901 87 Umeå, Sweden; 4 Department of Applied Physics and Electronics, Umeå University, 901 87 Umeå, Sweden; 5 Umeå Center for Functional Brain Imaging (UFBI), Umeå University, 901 87 Umeå, Sweden; 6 Umeå Center for Microbial Research (UCMR), Umeå University, 901 87 Umeå, Sweden

## Abstract

Visualizing medical images from patients as physical 3D models (phantom models) have many roles in the medical field, from education to preclinical preparation and clinical research. However, current phantom models are generally generic, expensive, and time-consuming to fabricate. Thus, there is a need for a cost- and time-efficient pipeline from medical imaging to patient-specific phantom models. In this work, we present a method for creating complex 3D sacrificial molds using an off-the-shelf water-soluble resin and a low-cost desktop 3D printer. This enables us to recreate parts of the cerebral arterial tree as a full-scale phantom model ($10\times 6\times 4$ cm) in transparent silicone rubber (polydimethylsiloxane, PDMS) from computed tomography angiography images (CTA). We analyzed the model with magnetic resonance imaging (MRI) and compared it with the patient data. The results show good agreement and smooth surfaces for the arteries. We also evaluate our method by looking at its capability to reproduce 1 mm channels and sharp corners. We found that round shapes are well reproduced, whereas sharp features show some divergence. Our method can fabricate a patient-specific phantom model with less than 2 h of total labor time and at a low fabrication cost.

## Introduction

Phantom models recreated from medical images have many potential roles in the field of personalized medicine. Reproducing patients’ internal structures as full-scale 3D models is beneficial for several reasons. First, phantom models can be used by medical professionals before surgical interventions, as well as for educational training^1–4^. Second, realistic phantom models in which flow properties can be measured could aid in disease diagnosis and provide researchers with a technique for evaluating computational fluid dynamics (CFD) simulations^5,6^. One example of a complex geometry with fine structured features that would be beneficial to recreate as a phantom flow model is the brain arteries. Recreating the cerebral arterial tree as a patient-specific model has the potential to help visualize and explore blood pressure effects from abnormal narrowing of blood vessels (stenosis) and validate current methods for clinical assessment^7^. However, current phantom models used in medicine are generally generic, expensive, and time-consuming to fabricate^8^. To overcome these limitations and fabricate patient-specific models, there is a need for a cost- and time-efficient pipeline from medical imaging to 3D phantom model.

Medical imaging has developed fast during the last decades with improved resolution of arterial angiograms (imaging of blood vessels) that are readily available from clinical routine measurements. However, turning these angiograms into phantom models as a part of the routine procedure, would require efficient fabrication methods. The recent development in 3D printer technology provides new ways of creating 3D models. As a result, phantom models can now be manufactured in-house, quickly and at a low cost. Most 3D printing technologies are either extrusion-based (e.g., FDM), stereolithography (e.g., SLA), or Inkjet printers (e.g., polyjet)^9,10^. The SLA printers are generally preferred due to their combination of high resolution, good surface finish and low cost. While all of these are important, the phantom model should also be able to resemble *in vivo* conditions, so its material must be considered. A silicone rubber called polydimethylsiloxane (PDMS) is commonly used in phantom models since it is transparent, inert, non-toxic^11,12^ and has an elasticity that can be tuned^13,14^. This is ideal for flow models that are used to conduct biological studies and/or incorporate compliant (flexible) elements. However, PDMS is not a material suitable for direct 3D printing since attempts result in degraded optical clarity^15^. Therefore, to allow for high optical clarity and high resolution, we propose a method using an SLA printer to fabricate a mold of the arteries and casting them in PDMS.

In the mold-based approach, the 3D printed scaffold (mold) occupies the space where the fluid will be and must be removed before the phantom model can be used. To do this, there are two main methods; the peel-away method and the sacrificial-mold method. The peel-away method allows for 2D or stacked 2D structures^16–18^ and some limited cases of simplified 3D geometries^19^. In addition, the sacrificial-mold method allows for fully 3D structures, only limited by the printing technology and the possibility to dissolve the scaffold. Both methods are successfully being used with molds produced by FDM printers, because of the availability of printing materials that are suitable for dissolving^20–22^. However, FDM printers are limited in how complex 3D structures they can produce, and achieving the smooth surface finish needed to validate flow simulations is difficult. SLA printers are therefore a better option, but 3D resins used by SLA printers are notoriously difficult to dissolve post curing and this has left us with only the peel-away method^23,24^, until now. Recent developments in commercial photopolymer resins have resulted in a water-soluble resin (IM-HT-WS, 3Dresyns) for SLA printers.

In this work, we propose a method that uses water-soluble resin to fabricate patient-specific phantom models in PDMS, using the sacrificial-mold method. This provides us with the ability to create flexible and transparent channel networks in 3D, not previously possible with an SLA printer. It paves the way for flow models with more complex shapes and better surface properties than what is allowed by FDM printers^25^, but at a much lower cost than Inkjet printers^26,27^. Our method can be used to develop these models for more advanced analysis, where the cost-effective and simple manufacturing process would be suitable for medical applications. We therefore test its ability to reproduce parts of a patient-specific cerebral arterial tree as a full-scaled phantom model in PDMS. We use magnetic resonance imaging (MRI) to compare the phantom model with the original patient data. Finally, we evaluate the accuracy and precision of the proposed method by fabricating simpler test channels.

## Results and discussion

### Recreating the arterial network in PDMS

**Figure 1:**
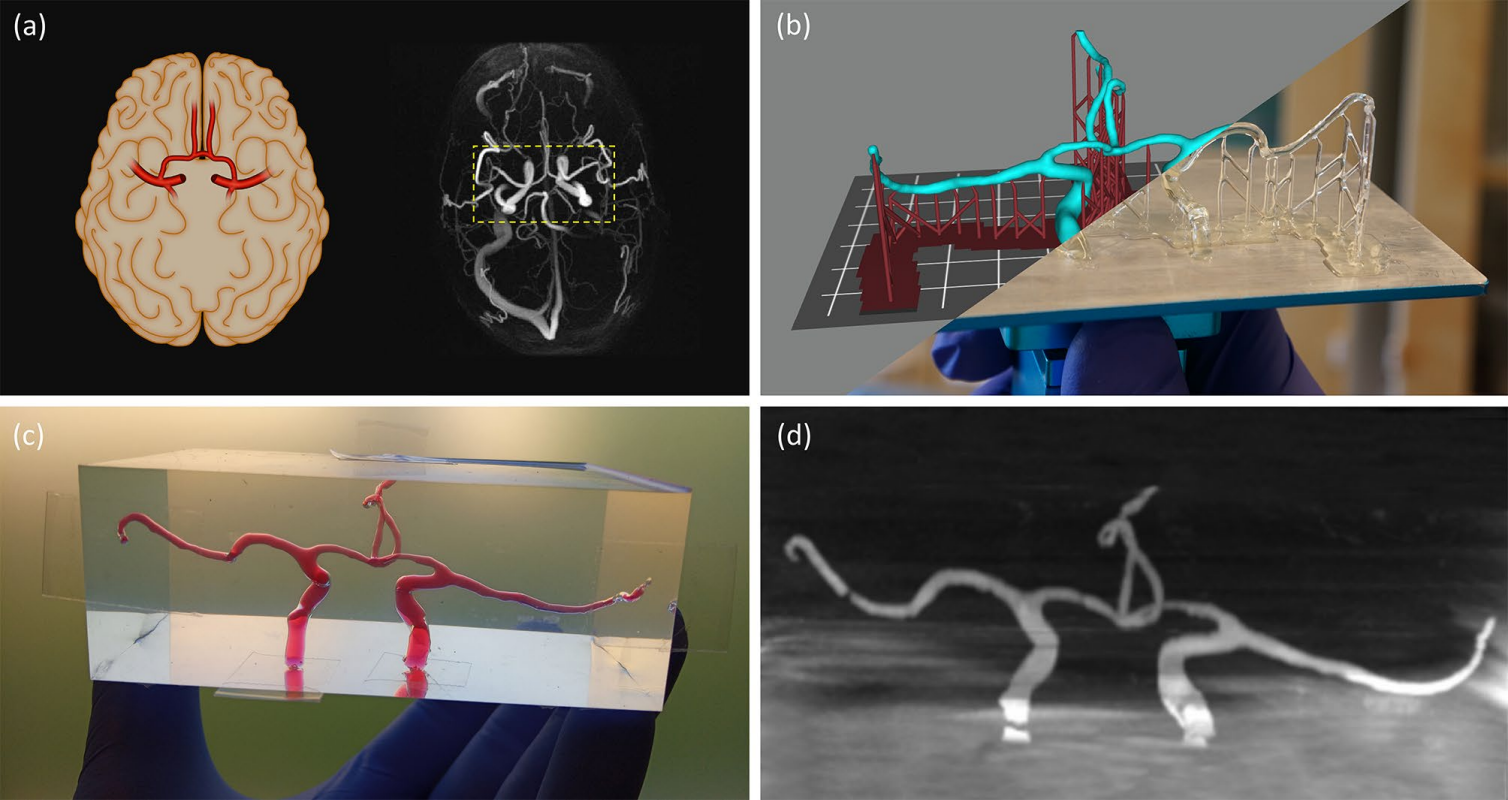
We recreated the anterior part of the cerebral arterial network as a full-scale phantom flow model in PDMS. This process consists of (**a**) creating a 3D model of the *circle of Willis* of a patient using CTA scanning techniques, (**b**) slicing and 3D printing a sacrificial mold using water-soluble resin, (**c**) casting PDMS around the scaffold before dissolving it, and (**d**) evaluating the flow channels using MRI. In panel (**a**), we illustrate the internal carotid, anterior and middle cerebral arteries and their position in the human brain (left), as well as show a maximum intensity projection from the 4D flow MRI angiogram of the patient (right)^28^, both as axial views. Panel (**b**) shows overlapping images of the arterial network after it has completed slicing (upper left) and printing (lower right), along with the temporary support structure. In panel (**c**), the finished PDMS model can be seen filled with a blood like substance (water and food dye) and with tape covering the channel-ends. An illusion of air bubbles can be seen and this is caused by total internal reflection for extreme angles. Finally, panel (**d**) show an MRI scan (coronal view) of our phantom model, before it is mapped in 3D and compared with the original model. In this case, some small air bubbles where actually present and these resemble channel restrictions in the MRI data.

To recreate the cerebral arteries of a patient as a full-scale phantom model, we used computed tomography angiography (CTA) data from a patient included in a research study conducted on symptomatic carotid stenosis patients with resulting ischemic stroke or transient ischemic attacks^28^. From the CTA images, we segmented the primary collateral pathway in the cerebral circulation, a part of the so called *circle of Wills* (see “Methods” section). In Fig. 1a, we show a schematic view of the internal carotid, anterior and middle cerebral arteries and their position in the human brain (left). The posterior circulation was not considered here. We also show a maximum intensity projection of a 4D flow MRI angiogram of the human brain (right), with the *circle of Wills* indicated by a yellow box (dashed). Subsequently, in Fig. 1b–d, we show each stage from patient- to phantom-model. This process consists of (b) preparing and printing the model, (c) molding and dissolving it in PDMS, and (d) evaluating the resulting flow channels with MRI.

#### 3D printing the sacrificial mold using an SLA printer

The principle of operation for an SLA printer is to build the part layer-by-layer, inside of a transparent vat filled with photosensitive polymer resin. After each exposure is completed, the build platform is raised slightly, allowing uncured resin to fill the next layer. This process is repeated until the part is finished. Prior to printing, we transform the 3D patient (CTA) model into machine code for the SLA printer using a computer program (Photon Workshop V2.1.24.RC7, Shenzhen Anycubic Technology Co. Ltd.) that divides the model into thin layers in a process called slicing. An exposure pattern (mask) is generated for each layer, along with its exposure time and other settings (see Fig. S3). To print our CTA model we used a desktop SLA printer (Photon S, Shenzhen Anycubic Technology Co. Ltd.) that cost less than $\$200$. It uses a liquid crystal display (LCD) as the mask, which ultraviolet (UV) light (405 nm) shines through. This technology is therefore also called mask stereolithography (MSLA) or digital light processing (DLP). In addition, our printer is of the inverted kind, which means that it builds the part upside-down. The minimum feature size and quality of the 3D printed part is defined by both the printer and the resin. The resolution of the printer is given by the pixel size of the LCD element and the minimum layer height of the build plate assembly, specified to be $47\,\upmu {\text {m}}\;x\;47\,\upmu {\text {m}}$ and $25\,\upmu {\text {m}}$, respectively. The photosensitive polymer resin we use is a newly developed water-soluble resin (IM-HT-WS, 3Dresyns) with properties suitable for the molding process, like high-temperature resistance (see Table S1). The formulation of the resin has been customized for our printer by the manufacturer, but it is possible to further optimize the resin by using additives. The printer uses $4.4\,ml$ resin when printing the 3D model and its support structure, at a cost of less than $\$2$.

#### Printer settings

Printing complex shapes often require a support structure, regardless of the printing technology. This is added during the slicing process to minimize flex during printing. However, the amount of support needed can be reduced by orienting the part correctly on the build plate. This should be done to avoid large overhangs while also averaging out the size of the exposure area between layers (large areas put higher stress on the underlying structure). Additionally, raising the part slightly from the build plate can be done to ease the removal of the part when printing is completed. With an SLA printer, the support structure is made from the same material as the finished part and must be removed manually before molding the PDMS model. Our support structure for the arterial network can be seen in Fig. 1b as small pillars with a sharp tip touching the part (dark red color). When setting up a printer for a new resin type, the most important parameter to optimize is the exposure time. This depends on both the printer and the resin, as well as the type of geometry that is printed. Long exposures will tend to clog up holes and channels, while short exposures will make thin pillars and walls unstable and deform. It is possible to quickly estimate a good starting point by using the coverslip method (see “Methods” section). In our case, the best results were attained for long exposures of 18 s and at a layer height of $50\,\upmu {\text {m}}$. To assure a good bonding with the build plate, the first couple of layers are usually overexposed (a brim might be added) and we expose the two first layers for 45 s each.

#### Post processing

After the printing process is complete ($\sim 5$ h), the part needs to be washed and cured. Before doing this, we remove the support pillars using some flush side-cutting pliers. A special solvent (Cleaning Fluid WS1, 3Dresyns) is used to wash away uncured resin from the surface. This also removes printing artifacts, but it must be done quickly ($<1$ min) to not over-dissolve the surface. Because of this, we use a soft brush to speed up the washing process. After that, we rinsed the part in acetone and submerge it in a second liquid (Cleaning Fluid WS2 Bio, 3Dresyns). Still submerged, we place the part under a UV LED light (15 W @ $400\pm 10$ nm FWHM) for approx. $15\,min$ and rotate it a couple of times to give an even exposure. Before the part can be used as a scaffold to mold our phantom model, we dry it with compressed air and store it overnight in an oven (60$^{\circ }$C).

#### Molding the phantom model

The 3D printed sacrificial scaffold is only the internal part of the mold, which later will be the channels of the phantom model, but we also need an outer container. For this, we made an open-top box ($L105\times W60\times H40$ mm, internal dimensions), designed to break apart when removing the cured PDMS and be reusable. The box is made from Plexiglas (*T*10 mm) to give a good surface finish on the outside of the model. Our 3D printed scaffold is then placed into the box, ready for molding. The PDMS (SYLGARD 184, Dow Corning) comes in two parts and we mix the elastomer base (Part A) with the curing agent (Part B) at a weight ratio of 10:1. To remove any air introduced during mixing, the PDMS is degassed ($\sim 10$ min) using a vacuum desiccator before we pour it into the box. It takes about 250 g PDMS to fill the box. The PDMS is then degassed ($\sim 5$ min) one more time and placed in an oven (2 h @ 80 $^{\circ }$C) to speed up the curing process. The mixing ratio, curing time, and curing temperature are variables known to affect the modulus of elasticity (Young’s modulus) of the cured PDMS. With our scheme, we measured a value of $2.31[3]\,MPa$ ($95\%$ CI) for the cured PDMS, using the compression gauge method (see “Methods” section). The phantom model is then removed from the box and holes ($\varnothing 1\,mm$) are made at every channel-end so that water can reach the sacrificial scaffold inside. To remove the water-soluble resin faster, we use an ultrasonic cleaner (Sonorex RK 31, BANDELIN electronic) and deionized water. Within $3\,h$ the channels are clear, but to be safe, we exchange the water and let it sit overnight. Since PDMS is slightly water permeable, we finally dry the model in the oven (60 $^{\circ }$C) and store it with desiccants.

### Test and evaluation of the phantom model

**Figure 2:**
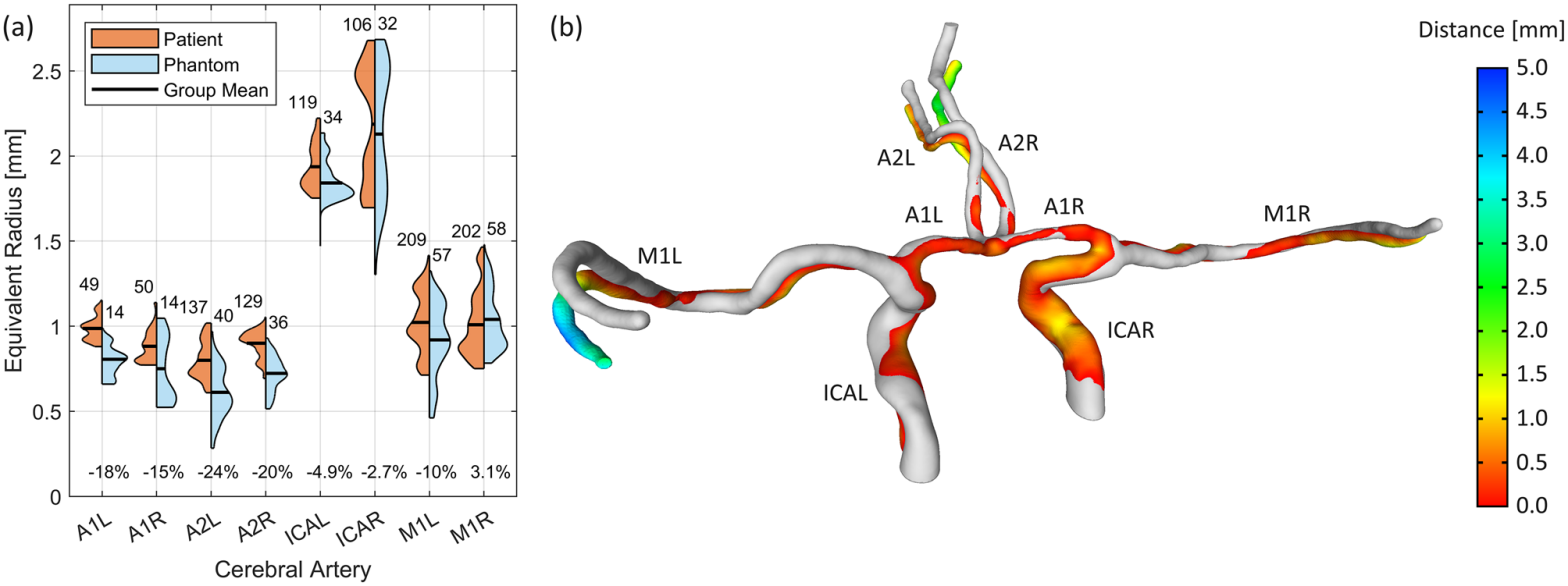
We compare the arterial network before and after recreating it as a phantom model in PDMS. In panel (**a**), we show the distributions of best fit circle radius along the arterial network of both the patient (CTA) model and the MRI scan of the phantom model. It is calculated at arbitrary positions (uniformly spaced) along each channel segment and the sample size is indicated above each group. The change in mean radius is shown in percentages for each segment and this is calculated relative to the CTA model. In panel (**b**), the patient model (gray) is superposed with the phantom model (colored) and a measurement of the channels deviation (Hausdorff distances) is shown by the color scale. *A* anterior cerebral artery, *M* middle cerebral artery, *ICA* internal carotid artery (*L* left, *R* right).

By printing the arterial network, molding it in PDMS, and dissolving the scaffold, we have created a full-scale patient-specific phantom model. We analyzed the model using non-destructive methods, so that it can be used for future experiments. First, we measured the internal volume of the phantom model to compare it to the CTA model. This was done by sealing the channel-ends with tape and filling it with water (and food dye) using a high-precision syringe, as seen in Fig. 1c. A volume of 1.40  ml was measured and compared with the CTA model, which is 1.61 ml. Then, with the phantom model filled with water, we used an MRI scanner (Discovery MR750 3.0T, GE Healthcare) to volume map the arterial network (see Fig. 1d) at a resolution of $390\,\upmu {\text {m}}\;x\;390\,\upmu {\text {m}}$ in the plane and $200\,\upmu {\text {m}}$ in height. The initial volume measurement helped us select a suitable threshold when transforming the stacked MRI images into a 3D model (i.e., chosen so that the total volume was 1.40 ml). In Fig. 2a, we divided the model into 8 arterial segments and compared the channel profiles between the CTA and the phantom model. This was done by looking at the radii distribution of best fitted circles, uniformly spaced along each segment. The phantom model showed a reduction in mean radii for most segments, with an average error of $-9.8\%$ for the whole model. However, we noted during the MRI measurement that some of the outliers (e.g., in A2L and M1L) were caused by small air bubbles trapped in the channels, which registered as false restrictions (see Fig. 1c,d). In Fig. 2b, we compare the CTA model (gray) and the phantom model (colored) by analyzing its deviation (Hausdorff distances) from the original CTA model. Some channels have bent downwards slightly during the manufacturing process, but this should not affect the flow characteristics of the channels.

### Further testing on straight channels

**Figure 3:**
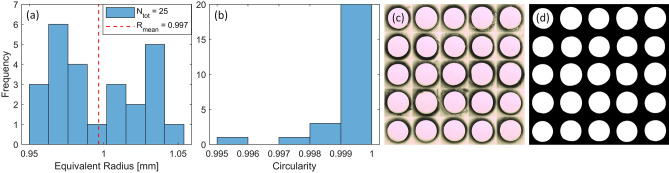
We examine the circular test channels with a design radius of $1\,mm$ using a microscope and analyze them for size and shape in MATLAB [R2021a, The MathWorks Inc.]. This was done by 3D printing, molding and dissolving 25 individual channels, cutting a cross-section from each, and photographing them. Panel (**a**) shows the distribution of equivalent radius and this is calculated as the radius of a circle with the same area as the channels cross-section. In panel (**b**), we show an estimate of the channels circularity. It is calculated as $(4\pi \,Area) / Perimeter^2$, and approach unity for a perfect disk. In panels (**c**) and (**d**), a montage of all the cross sections are shown before and after image filtering, respectively.

To better test the reproducibility of our method we also made some simple straight channels. For this, we printed 25 pillars with a circular cross-section of $1\,mm$ in radius. These pillars were printed perpendicular to the build plate, molded in PDMS, and dissolved. In contrast to the arterial model, no support structure was required here and as the outer container, we used a plastic petri dish ($\varnothing 35\times H10$  mm, internal dimensions). We then cut the channels into thin slices and imaged them with a spatially calibrated microscope (MICROPHOT-FX, Nikon Corporation), at a resolution of $1.5\,\upmu {\text {m}}\;x\;1.5\,\upmu {\text {m}}$. We used an in-house developed image analysis program (MATLAB R2021a, The MathWorks Inc.) to estimate the equivalent radius of the cross-sections, as well as their circularity. Figure 3a shows the distribution of the channels radii and a mean equivalent radius of $0.997\pm 0.061$ mm ($95\%$ CI), which is very close to the design radius but with some noticeable spread. Figure 3b shows an estimate of the channels circularity and these are close to unity for most channels, indicating a near perfect circular shape and a smooth interior surface of the channels. In Fig. 3c we show a montage of all 25 cross-sections upon imaging and in Fig. 3d, we show their shapes at the time of analysis (after filtering). We also printed similar channels with a square cross-section to investigate how well the printer can reproduce these. Here, we observed a rounding effect of sharp corners (see Fig. S2). However, this should not affect our main aim since sharp features are rare on the arterial network and other biological structures.

### Applications of 3D printed in vitro models

Since the presented method can produce phantom models in silicone rubber of different compositions, material properties like elasticity, index of refraction, and transparency can be tuned. The optical clarity of a PDMS model allows for visualization of anatomical structures. It also enables flow imaging techniques that require refraction index matching, like particle image velocimetry^29^. This, combined with syringe/peristaltic pumps and integrated pressure sensors, make a complete setup for *in vitro* experiments of patient-specific arteries, which could be used to verify CFD simulations^28^. In this case, flow conditions can also be advanced beyond what is possible to observe in clinical settings, simulating effects on perfusion pressure of an increased degree of stenosis and helping to find stress points^30^. In this work, we used MRI and microscopes to analyze the phantom model, but these materials are also compatible with pulsed photothermal radiometry, ultrasound, and more^31,32^.

**Figure 4:**
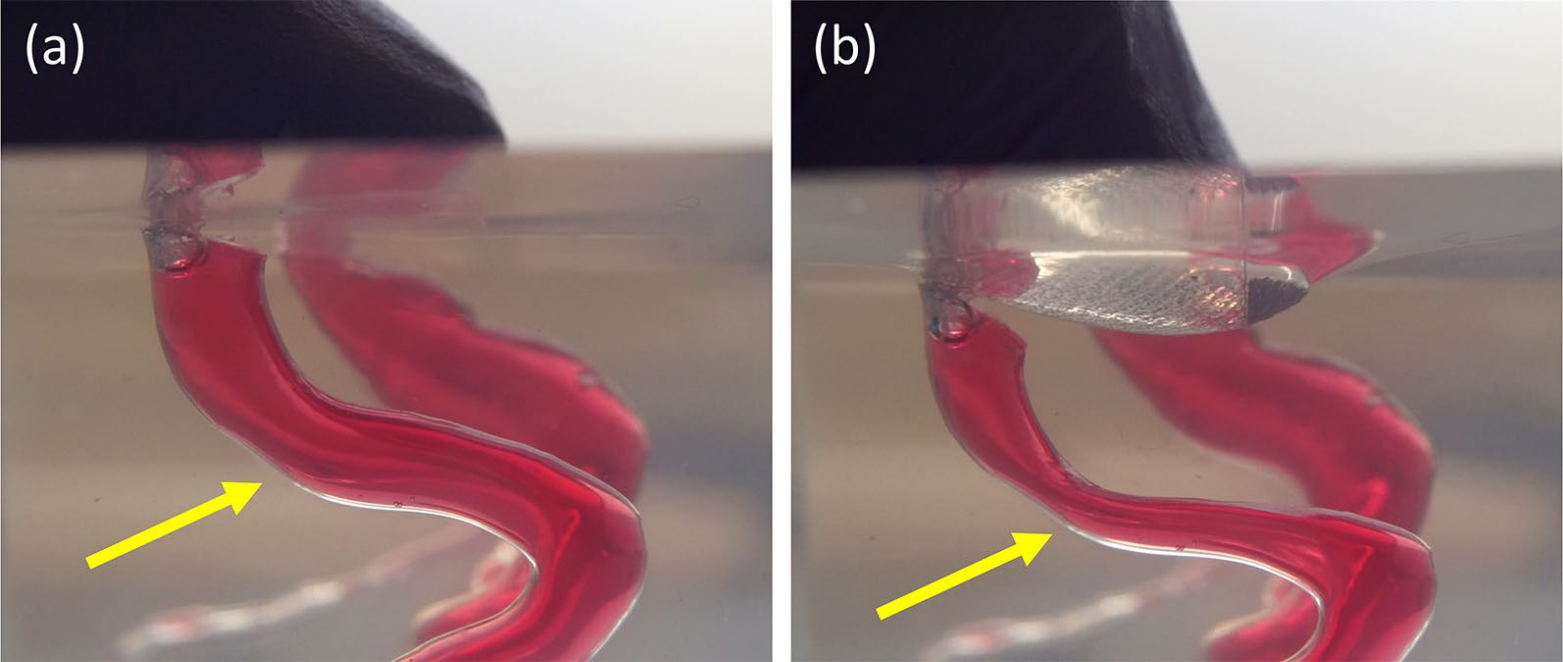
PDMS has a flexible characteristic that can mimic real blood vessels and is therefore used in the phantom model. In panel (**a**) and (**b**), we show the effect on the model before and during the surface is depressed with a thumb, respectively. The channels are filled with a blood like substance (water and food dye) and an external force reduces the size of the channel significantly (as indicated by arrows). Showing possible applications of these compliant phantom models, for example, as to simulate flow restrictions in blood vessels.

The flexible nature of these silicone rubbers is demonstrated in Fig. 4 and this allows us to construct phantom flow models that better mimic *in vivo* fluid-to-wall interactions, or control channel shape and size using compliant mechanisms. Elastic walls also allow for realistic models in which pulse wave propagation^33^ can be experimentally determined and compared to observations. Pulse wave propagation is of interest in the context of atherosclerosis^34,35^, the major mechanism behind stroke and cardiac attacks^36^. Atherosclerosis causes stiffening of arterial walls, increasing the arterial pulse amplitude and reducing dampening as the pulse wave travels towards the capillaries^37^. The atherosclerotic process further increases the risk for arterial wall plaques buildup that can rupture and occlude critical downstream blood vessels. Measurements of such pulse wave propagation are frequently used to estimate vascular stiffness indirectly^33^, but the lack of a reliable reference is imposing challenges on such methods as measurement accuracy cannot be established. Another application is within vascular surgery, where it is sometimes necessary to turn off the carotid artery during surgery^30^. A phantom model that is quick and easy to fabricate provides the opportunity for bench testing in surgical planning to identify patients at risk of hypoperfusion when the carotid is closed.

Moreover, flexible phantom models could also help in investigating the recently discovered glymphatic system. This is a system that clears metabolic waste products from the brain by pumping cerebrospinal fluid through the brain parenchyma^38^. A dys-functioning glymphatic system can lead to degenerative diseases, like Alzheimer’s, and it is proposed that the flow is driven by pulsatile arterial wall movements^39^. With our method, it is possible to test this claim by adding a periarterial compartment around the arteries and investigating the significance of arterial wall movement in the glymphatic system.

### Production cost and labor time

The consumption costs per full-scale phantom model is around $\$70$ and it consists mainly of the cost for the PDMS (SYLGARD 184, Dow Corning). To reduce the cost, we tried molding the same CTA model in a less expensive silicone rubber (T-30, PS Composites). This reduced the overall costs to only $\$14$ per model, but gave a lower optical clarity (see Fig. S4). For completeness, we also measured the volume of this phantom model to 1.63 ml, which is much closer to the volume of the CTA model. This improvement is contributed to a better tuned print and washing process of the scaffold. Moreover, the total time from patient- to phantom-model is around two days. Fortunately, all time-consuming steps can be done unsupervised which results in less than 2 h of total labor time to create a phantom model, excluding imaging and segmentation of CTA data.

## Conclusion

In this work, we developed a cost- and time-efficient pipeline: from medical imaging (CTA) to full-scale 3D phantom model. For this, we used a simple SLA 3D printer that costs less than $\$200$ to print water-soluble scaffolds of brain arteries. We molded the scaffolds in flexible silicone rubber (PDMS) and dissolved it in water (see Fig. 1). We then compared the flow channels of the phantom model with the arteries in the CTA model (see Fig. 2) and found good agreement between the two. The phantom model shows a $13\%$ reduction in total volume and by looking at cross-sections along the arteries, we noted a decrease in radius for the narrower channels. On the contrary, the trial with a less expensive silicone (T-30) resulted in a phantom model with a volume $1\%$ larger than the CTA model.

To further assess the accuracy and precision of the process, we also fabricated circular test channels of $1\,mm$ in radius (see Fig. 3). All of them show high circularity and smoothness for the inner walls, which is important for flow models. Here, we also noted a spread in the channel radius of $\pm 6\%$ ($95\%$ CI), similar to the phantom models. Again, we attribute this discrepancy to the printing/washing process and suggest the use of additives for fine-tuning the optical density of the resin to the printer (UV light source) and chosen printing depth. This would help minimizes stray light and enhance the precision, especially for small features. New variants of water-soluble 3D resins have also been introduced since we started this work. We believe that this, in conjunction with using a more sophisticated 3D printer, will enable phantom models with higher accuracy and reproducibility in the near future.

In conclusion, the newly developed water-soluble resin for 3D printers has provided a quick and easy way to fabricate patient-specific phantom models in flexible and transparent silicone rubber. These have suitable properties for a range of applications. For example, for medical education, the ability to rapidly produce cheap phantom models is an important step forward both in theoretical teaching (visualization) and for practical (rehearsal) training. Also, these phantom models can be used for training in MRI/CT imaging or Doppler ultrasound measurements. Finally, phantom models can help with dynamic flow simulations in clinical neuroscience as well as in personalized medicine.

## Methods

### 3D printing sacrificial molds using water-soluble resin

Here we summarize a step-by-step guide for creating flow models in PDMS using a 3D printer and water-soluble resin.

- **1. Modeling**-Prepare a 3D model with the internal geometry of the phantom model.
- **2. Slicing**-Slice the part with suitable settings and generate the support structure.
- **3. Printing**-Prepare the SLA printer and print the part using the *IM-HT-WS* water-soluble resin.
- **4. Detaching**-Separate the part from the build plate and remove the support structure (use side-cutters if needed).
- **5. Washing**-Wash away uncured resin ($<1$  min) with cleaning fluid *WS1* and a soft brush, followed by a rinse in acetone.
- **6. Curing**-Expose the part to UV light for approx. $15\,min$ (at $400\,nm$) while it is immersed in cleaning fluid *WS2*.
- **7. Drying**-Dry the part with compressed air and leave it in the oven over night ($60^{\circ }C$).
- **8. Mixing**-Mix the base and curing agent at a weight ratio of 10:1.
- **9. Degassing**-Remove air bubbles from the PDMS with a vacuum desiccator.
- **10. Molding**-Place the part into a suitable container and pour in the PDMS mixture (redo step **9** if needed).
- **11. Hardening**-Place the container in the oven ($2\,h$ @ $80^{\circ }C$) to let the PDMS cure.
- **12. Preparing**-Remove the phantom model from the container and punch holes for each channel-end.
- **13. Sonicating**-Dissolve the internal structure in deionized water using a ultrasonic cleaner.
- **14. Storing**-Dry the phantom model in the oven ($60^{\circ }C$) and store it with desiccants to remove any moisture.

#### Segmenting the CTA patient data

The patient-specific geometry was obtained from a clinical CTA investigation from a patient included in a larger study^28^. The Ethical review board of Umeå University and the Swedish Ethical Review Authority (Dnr: 2011-440-31M; Dnr: 2019-05909) approved the study. It was performed in accordance with the guidelines of the Declaration of Helsinki. Oral and written information about the study was given to the participant and written informed consent was obtained from the participant. The segmentation was performed with Synopsys’ Simpleware$^{TM}$ software (ScanIP P-2019.09, Synopsys, Inc., Mountain View, USA) and the exported CAD generated with the Simpleware FE module. The original image data had a resolution of $510\,\times \,500\,\upmu$m in the trans-axial plane, and a slice thickness of $400\,\upmu$m. The image data originally covered the entire cranium but was before the segmentation cropped to only include the cerebral arteries of interest. The image volume was thereafter resampled with linear interpolation to obtain an isotropic resolution of $300\,\upmu$m. Before segmentation, we used an edge-preserving bilateral filter to reduce background noise from surrounding tissue. We only analysed the anterior part of the circle of Willis, as motivated in the previous study^28^. A coarse segmentation of the cerebral arterial tree was extracted by a threshold filter, from which the software specific gradient-based filter ’Local surface correction’ was used for wall detection. This filter used the image background intensity to adjust the segmented surface. Since we were only interested in the major arteries, smaller branches were manually removed, in addition to remaining parts of the scull-bone. The segmentation was finalized by applying a volume and topology smoothing filter on the mask. The CAD-file was exported with a target maximum and minimum element size of $600\,\mu m$ and $300\,\upmu$m, respectively, aiming for the interpolated image resolution.

#### Estimate exposure time: the coverslip method

To get a quick estimate of the exposure time needed for a particular resin and printer, you can use the cover slip method. This is done by putting a drop of the photosensitive polymer resin on a glass microscope cover slip, placing it on the projection screen of the printer and manually exposing it (see the settings menu on the printer). After that, wash away uncured resin with a suitable solvent and a soft brush. Measure the relative thickness of the cured resin by using a micrometer screw gauge, or the like. Repeat this for different exposure times and at even increments until a thickness of about $150\%$ of the intended layer height is found. Additionally, there is a wide range of open-source calibration models online that can help you test the effects from exposure time on different kinds of features and further optimize our printer parameters.

#### Measure Young’s modulus: the compression gauge method

To measure the modulus of elasticity of our phantom model without having to cutting out a sample piece, we used the remaining PDMS mix to simultaneously make some small sample moldings ($\varnothing 8\, \times \, H6$ mm). The compression gauge method uses a custom-built compression instrument, made from a through-axis dial indicator. The sample cylinder is placed between a fixed test surface and the bottom-end of the indicator axis, where a flat shoe is mounted to distribute the force evenly over the whole sample cylinder. The strain on the sample is then increases by adding weights (in steps) at the top-end of the indicator axis. The stress deforms the sample and this is recorded from the dial indicator. The Young’s modulus is given by the slope of a linear regression between the stress and strain (see Fig. S1), as $E=\Delta \sigma /\Delta \epsilon$, and analyzed in MATLAB [R2021a, The MathWorks Inc.]. The stress is calculated as $\sigma =mg/A$, where *m* is the mass of the added weights, *g* is the gravitational acceleration, and *A* is the surface area of the sample. The strain is calculated as $\epsilon =L/L_0$, where *L* is the length decrease caused by deformation, and $L_0$ is the initial rest length of the sample. Because we look only at the slope, the initial weight of the setup will not affect the result, but more measuring points will increase its accuracy. However, linear deformation only occurs at low stress (below $\sim 25\%$) and this is where we calculated the Young’s modulus.

## Supplementary Information

Supplementary Information.
