# Supplementary material for: Patient-specific brain arteries molded as a flexible phantom model using 3D printed water-soluble resin
Source: Sci Rep. 2022 Jun 17;12:10172. doi: 10.1038/s41598-022-14279-7 (PMC9205921; doi:10.1038/s41598-022-14279-7)
Supplement: Supplementary file 1 — Supplementary Information. [file 41598_2022_14279_MOESM1_ESM.pdf]

Patient specific Brain Arteries Molded as a Flexible Phantom Model using 3D printed water-soluble resin

Daniel PG Nilsson[1], Madelene Holmgren[2,3], Petter Holmlund[2], Anders Wåhlin[2], Anders Eklund[2], Tobias Dahlberg[1], Krister Wiklund[1], Magnus Andersson[1,4,*].

[1]Department of Physics, Umeå University, 901 87, Sweden

[2]Dept. of Radiation Sciences, Radiation Physics, Biomedical Engineering, Umeå University, 901 87, Sweden

[3]Department of Clinical Science, Neurosciences, Umeå University, 901 87, Sweden

[4]Umeå Centre for Microbial Research (UCMR), Umeå University, 901 87, Sweden

*Corresponding author: magnus.andersson@umu.se

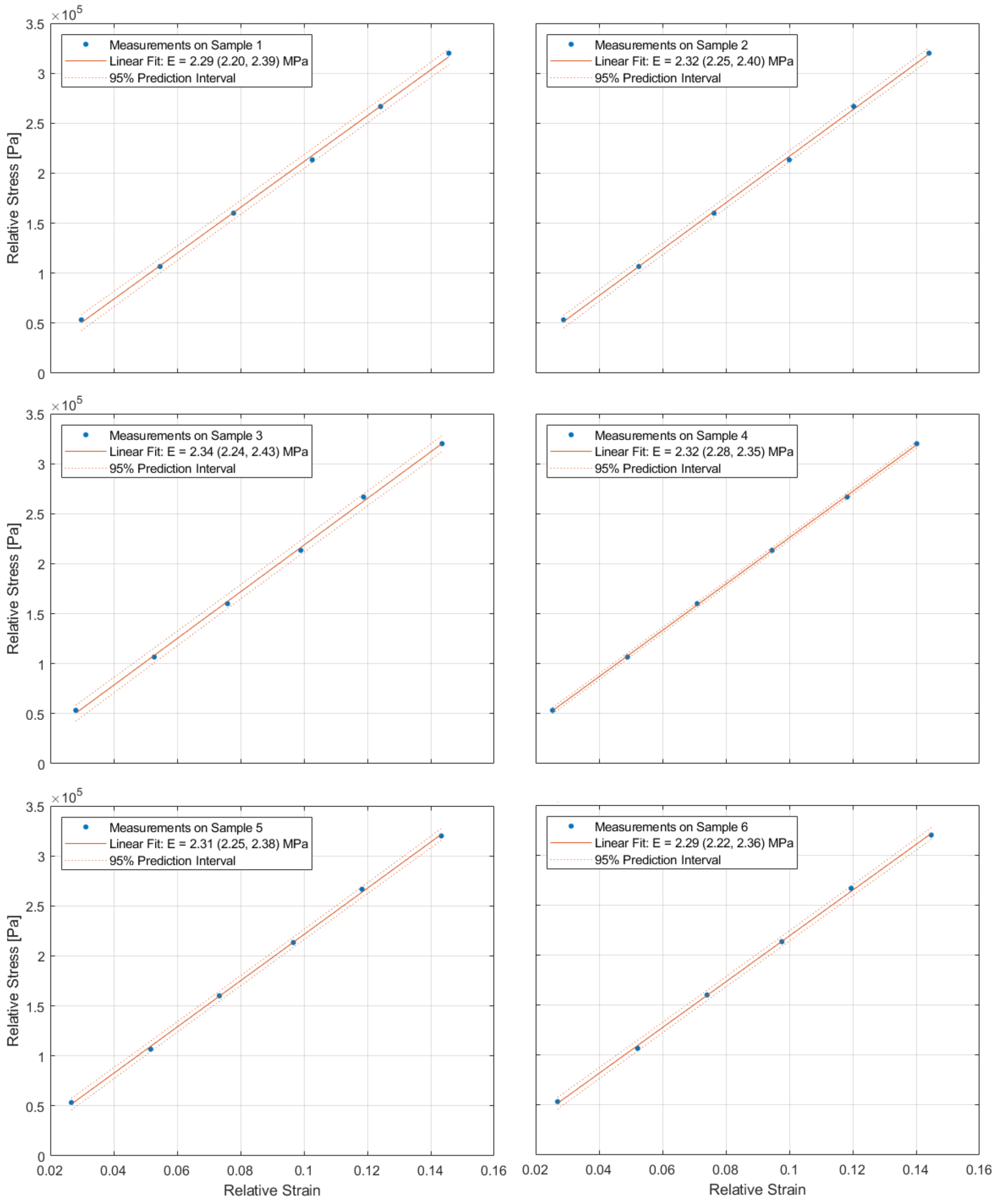

Figure S1. Compression measurements on six sample cylinders (Ø8xH6mm) made of PDMS [SYLGARD 184, Dow Corning] and conducted with the custom-built compression instrument (see figure S1). A linear regression is made between the Stress and Strain, which yields the modulus of elasticity (E) with a corresponding 95% confidence interval. The cylinders were prepared identically and cured under the same circumstances and should thus have similar properties. Combining these results gives a final value of E = 2.31 [3] MPa.

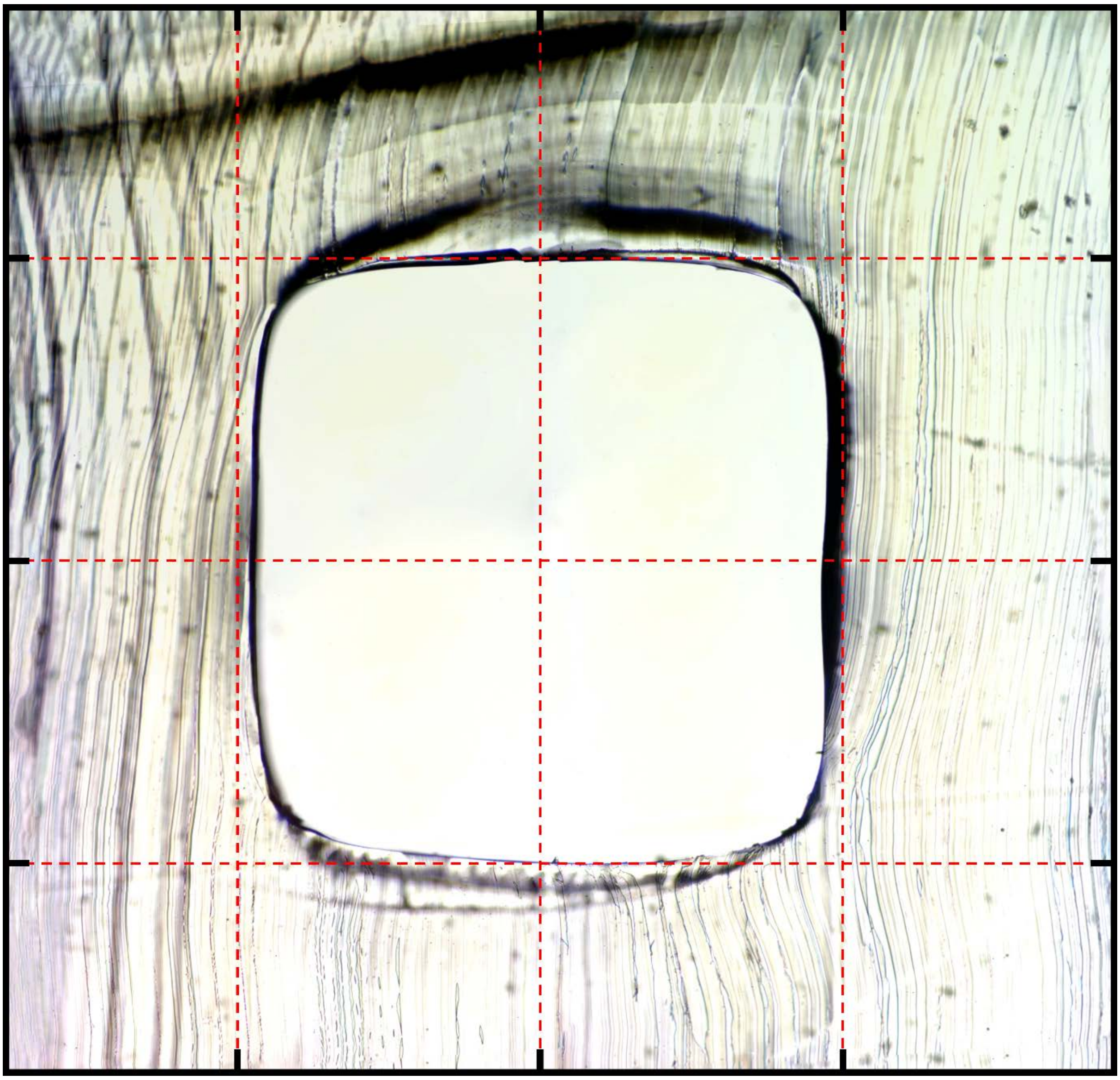

Figure S2. Picture of a slice that is cut from a flow channel in PDMS. The channel is designed to be straight, with a square cross section area (2x2 mm) and printed perpendicular with the build plate. It has been manufactured with our method of 3D printing sacrificial molds using water-soluble resin. The dashed (red) lines are spaced 1 mm apart.

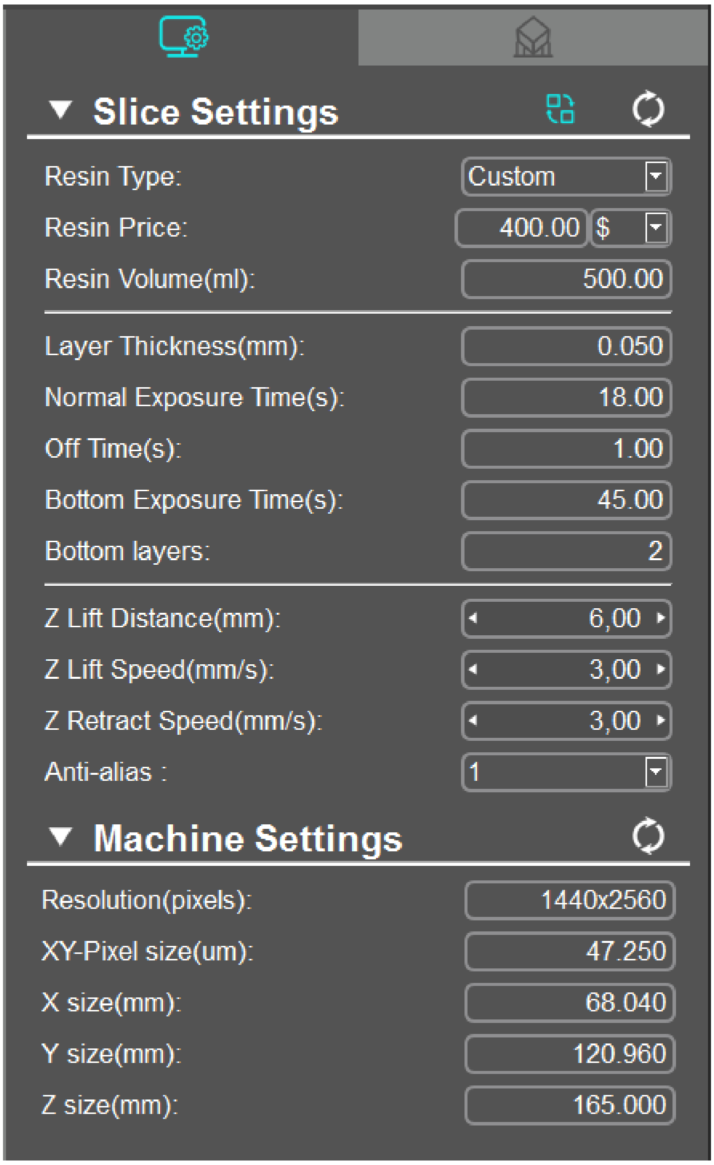

Figure S3. Settings for the slicer program (Photon Workshop V2.1.24.RC7, Shenzhen Anycubic Technology Co. Ltd.) used when printing with water-soluble resin (IM_HT_WS, 3Dresyn). Most important is the layer thickness, normal exposure time, bottom exposure time and the number of bottom layers. The machine settings represent the 3D printer model used (Photon S, Shenzhen Anycubic Technology Co. Ltd.).

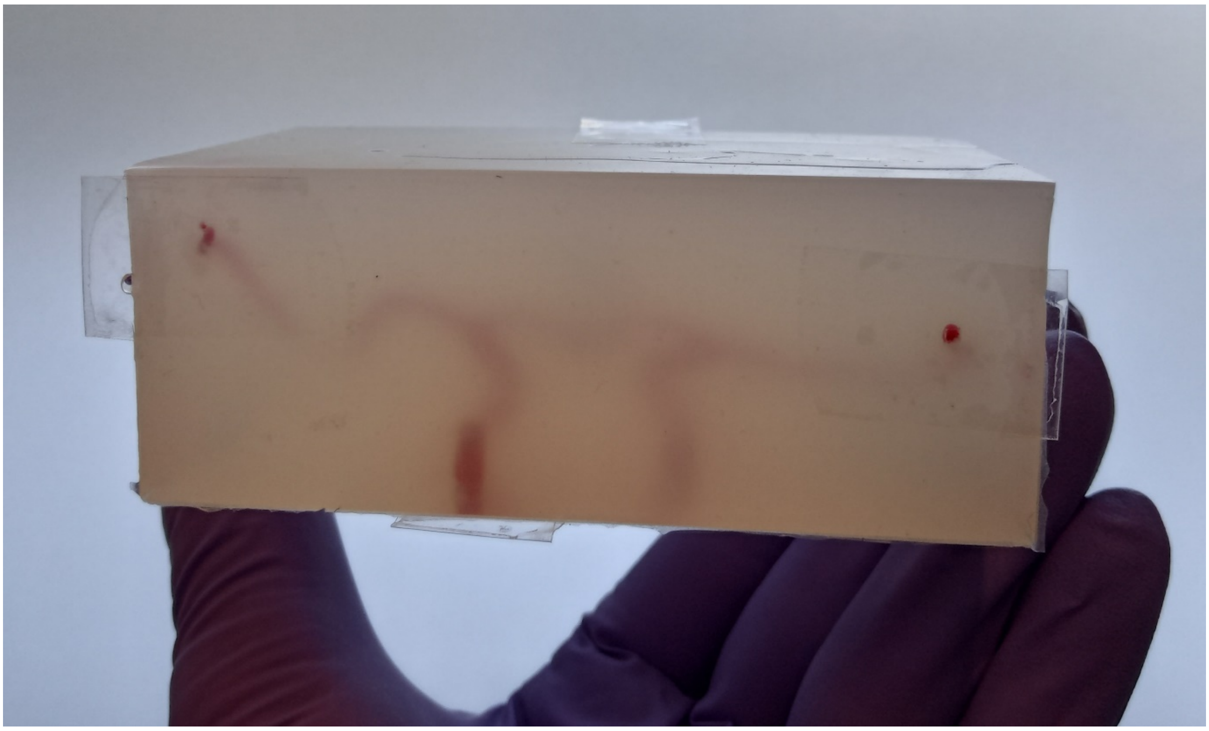

Figure S4. The arterial network was also molded in a less expensive silicone rubber (T-30, PS Composites), which has a lower optical clarity. Here it is also filled with a blood like substance, with tape covering the channel-ends. This shows a way of reducing the overall cost for manufacturing phantom models in cases where clarity is not desirable.

Table S1. 3Dresyn IM-HT-WS, hard and tough and water-soluble for sacrificial low \& medium temperature injection molding

- Recommended for printing Water-Soluble WS sacrificial Injection Molds IM for low and medium injection temperature materials, plastics, silicones, waxes, etc...
- Hard and tough HT, Shore Hardness D80
- Fast solubility in water
- Tack free finishes after light box postcuring
- Very high tensile strength >40 MPa
- High rigidity (Young modulus >2000 MPa) to permit printing of thin rigid \item mold walls to maximise solubility speed and minimum resin consumption
- Non brittle at injection temperatures
- Elongation <8\%
- Very low viscosity, below 100 cps
- High resolution once tuned to the printer specifications and after full workflow implementation
- Very low shrinkage
- Printable by most commercial and professional SLA, DLP \& LCD 3D printers
- Improved water solubility is achieved with low light power printers, such as standard LCD printers
- Fast printability with even low power LCD printers
- Increased durability of the resin tank
- Metal and organo-tin free
